# A Shave

**Published:** 1881-12

**Authors:** 


### A SHAVE.

He removed his coat, and, as he seated himself in the barber's chair, said, simply, "A shave."

"Yes, sir," answered the barber, pulling out a couple of towels, and whisking one across the customer's chest. "Rather a late fall, this, sir," he continued, as he drew his razor across a strap.

"Yes," said the customer.

"Heard anything about the Cabinet this morning, sir?" asked the barber, daubing a great blotch of lather on his customer's right cheek, and bringing the brush under the chin with an artistic flourish.

"No," said the customer, shutting his eyes.

"Now it's my opinion, sir," continued the barber, "that Arthur will make a fair President. What do you think about it?" and the barber drew the razor down his victim's face with alarming rapidity.

"Don't know," said the customer.

"Ever shave yourself, sir?" asked the barber.

"No," answered the victim, feeling that every time he moved his jaws he was risking his life.

"Didn't know but what you might shave yourself now and then. Razors handy to have in the house, sir; got any?"

"No," answered the victim.

"Now, I have a pair I can sell very cheap," said the barber; "show 'em to you in a moment. English make, sir."

"Don't want 'em," said the customer, squirming as a tender spot was struck.

"Hair's a little thick," said the barber, running his fingers through the customer's locks, and stopping short in his shaving.

"Yes," said the victim absently.

"Better let me trim it up a bit; won't take ten minutes," said the barber, slapping a wet towel across the customer's mouth, eyes and nose, and all but blinding him.

"Pish! foo! no!" said the customer.

"Better have your head washed, then. Brighten you up amazingly. Won't take five minutes. Here, Johnny, turn on that warm water."

"No, no!" said the customer, springing desperately from the chair.

"Clothes brushed, sir?" said Johnny, rushing up and commencing a vigorous attack upon the victim with an immense wisp broom.

"No-o-o!" and the customer bolted.

